# “Dead” Layer Dynamics of Poly(ethyl methacrylate) in Nanotubes

**DOI:** 10.1021/acsmacrolett.6c00291

**Published:** 2026-07-22

**Authors:** Jianwei Zhang, Jiajia Zhou, Hans-Jürgen Butt, George Floudas

**Affiliations:** † 28308 Max Planck Institute for Polymer Research, 55128 Mainz, Germany; ‡ Faculty of Materials Science and Engineering, 26467 South China University of Technology, Guangzhou 510640, China; § South China Advanced Institute for Soft Matter Science and Technology, School of Emergent Soft Matter, South China University of Technology, Guangzhou 510640, China; ∥ Department of Physics, University of Ioannina, 45110 Ioannina, Greece; ⊥ National-certified Enterprise Technology Center, Kingfa Science and Technology Co., Ltd., Guangzhou 510663, China

## Abstract

We report the segmental dynamics of poly­(*n*-ethyl methacrylate) melts forming nanotubes inside cylindrical alumina nanopores (AAOs). Long nanotubes were fabricated at the inner pore walls by the polymer precursor film during the complete wetting transition at high temperatures. Using differential scanning calorimetry and dielectric spectroscopy, we show that the segmental dynamics in the nanotubes are bimodal and slower than in bulk with a broad distribution of relaxation times. These features reflect the dynamic properties of the “dead layer” formed by the adsorbed polymer chains at the pore walls.

## Graphical abstract

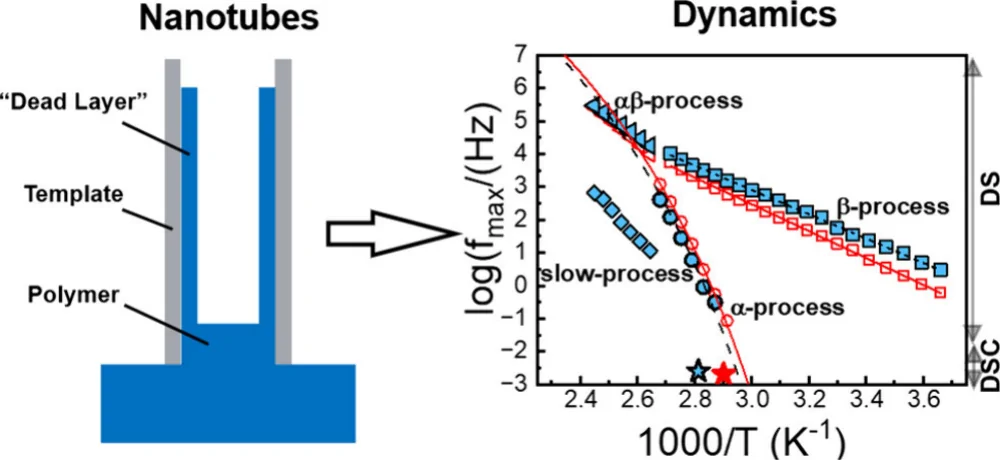

Despite the numerous applications of polymer wetting of solid substrates^1–4^ (including lubrication, coatings, adhesion, etc.), important aspects are not fully understood, including the pertinent polymer dynamics. A way to investigate the properties of wetting is by employing polymers as they penetrate narrow pores of high energy solid materials.^5–7^ Polymer imbibition in nanopores is described by the competition of two mechanisms.^8,9^ The first is the standard hydrodynamic flow, resulting in a parabolic flow profile. When the inner wall has a strong attraction to the polymer, a layer of immobile chains is created (called “dead layer”), resulting in an increase of the effective viscosity and to slower imbibition.^8^ The second is the chain reptation under a pressure gradient, leading to a plug flow profile and to the reduction in the effective viscosity (faster imbibition).^9^ For low molar mass polymers, the former mechanism dominates. When such polymers penetrate self-ordered nanoporous alumina templates (abbr. AAO), a “dead zone” is formed that causes deviations from the classical Lucas-Washburn equation^10,11^

1 $$h\left(t\right)={\left(\frac{\gamma R\cos\theta }{2\eta }\right)}^{1/2}\sqrt{t}$$

Here, *h*(*t*) is the imbibition length, γ is the surface tension, θ is the advancing contact angle, *R* is the pore radius, and *t* is the imbibition time. Importantly, the characteristic *t* ^1/2^ scaling remains unchanged; the deviation manifests solely in the prefactor, which no longer matches the Lucas-Washburn expression. One major question is on the segmental dynamics within the “dead layer”.^12–19^ Molecular dynamics simulations of polymer imbibition in nanopores have revealed that adsorption creates the “dead layer” with significantly reduced chain mobility, effectively reducing the pore radius.^20,21^ Investigating the dynamics within the dead layer is not an easy task. Simulations have an advantage in this respect as they can infer the segmental mobility as a function of the distance from the solid walls. Results have shown that the zone extends to 1–2 times the radius of gyration.^20–27^ On the other hand, experimentally this zone is not readily accessible.

Here, in order to investigate the polymer segmental dynamics within the “dead layer” we employ a different approach. We fabricate *nanotubes* by following the kinetics of the *wetting transition* of a polar polymer within cylindrical alumina nanopores. Earlier studies have shown that the wetting of the inner wall of AAO nanopores by a precursor film is a rapid process. It can wet the whole AAO surface.^5–7^ The wetting transition depends on several factors, including temperature, time, and polymer molar mass. In complete wetting, the precursor film spreads on the inner walls of the nanopores ahead of the main capillary front of the polymer melt giving rise to *nanotubes*. In partial wetting, nanopores are gradually filled with the polymer melt by capillary action giving rise to *nanorods*. Complete or partial wetting depends on the sign of the spreading coefficient, *S* = γ_SG_ – γ_SL_ – γ_LG_, where γ_SG_, γ_SL_, and γ_LG_ are the solid–gas, solid–liquid, and liquid–gas interfacial energies, respectively. When *S* ≥ 0 there is complete wetting, whereas *S* < 0 gives partial wetting of the surface.^1^

The spreading of the precursor film, driven by surface interactions, may lead to unique chain alignment or adsorption states at the polymer–template interface. These states could potentially “freeze” nonequilibrium conformations in the vicinity of the liquid-to-glass temperature, directly affecting the interfacial stability and mechanical integrity of the nanotubes.^28–35^ Hence, it is important to investigate the segmental dynamics of polymers that form nanotubes. For this study, we employ poly­(*n*-ethyl methacrylate)­s (PEMA) with two molar masses. PEMA is a polar polymer with accessible dynamics, as determined by dielectric spectroscopy over a broad temperature range.^36,37^ We first establish the conditions (temperature, time, and molar mass) leading to nanotube formation. Subsequently, we investigated the segmental dynamics with dielectric spectroscopy (DS) and differential scanning calorimetry (DSC). The results show that the “dead layer” is composed of segments with substantially slower segmental dynamics as compared to the bulk.

Two poly­(ethyl methacrylate) (PEMA) samples were used to fabricate nanotube structures. Their molecular characteristics (**PEMA-7k** and **PEMA-37k**, with respective *M*_n_ values of 7 and 37 kg/mol) are shown in Table S1. Self-ordered nanoporous aluminum oxide (AAO) templates with a pore diameter of 400 nm and a uniform pore depth of 100 μm were fabricated following established literature protocols (AAOC400).^5,6^ In addition to nanotubes, we investigate the segmental dynamics within another confining geometry. We used V-shaped AAOs with a top pore diameter of 450 nm, a bottom pore diameter of 100 nm, and pore depth of 2.5 μm, denoted as V-450-100-2500. The V-shaped templates have a pore depth of only 2.5 μm (as compared to 100 μm in the case of the cylindrically shaped nanopores) hence it is not possible to stabilize nanotubes with V-shaped nanopores. The templates have been characterized by different methods including SEM, FIB, AFM, and laser interferometry, as reported elsewhere.^5,6,17,28–35,38^

We systematically optimized the temperature and time parameters for the two PEMA homopolymer infiltrations using the template wetting method (see Figure S1, Supporting Information). First, a PEMA film was placed on top cylindrically shaped AAO templates with 400 nm diameter and 100 μm depth nanopores (AAOC400). The assembly was transferred to a preheated oven for vacuum annealing. The optimal temperature and wetting time for the formation of nanotubes were determined as *T* = 463 K (i.e., *T*_g_ + 128 K) and 5 min for **PEMA-7k**, and *T* = 493 K (i.e., *T*_g_ + 158 K) and 10 min for **PEMA-37k**, respectively. Subsequently, samples were rapidly cooled to room temperature to preserve the nanostructures. For the DS measurements the nanotubes were not released from alumina. For the SEM characterization, the AAO templates were dissolved using a 5 wt % NaOH aqueous solution, yielding free-standing PEMA nanotubes supported by the residual bulk film.

The polymer nanostructures were investigated by scanning electron microscopy (SEM-SU8000). Top-view and cross-sectional images of PEMA nanotubes at different heights were analyzed. Figure 1 and Figure S2 depict free-standing PEMA fibers for **PEMA-37k** and **PEMA7k**, respectively, following AAO removal. In some cases, the nanotubes broke at different height in the process of removing the AAO template. The thickness of the precursor film evidenced by the nanotube wall thickness in both cases was around 100 nm. This should be compared with the coil size. The radius of gyration (*R*_g_) of **PEMA-7k** and **PEMA-37k** was determined to be around 2.7 and 6.1 nm, respectively.^39^ Therefore, for **PEMA-37k** the nanotube thickness is approximately 15 × *R*_g_. Notably, this is the smallest *stable* nanotubes that could be prepared with PEMA. Using different temperature/imbibition time conditions (Figure S3) or smaller nanopores (Figure S4) resulted in *nanorods* instead of *nanotubes*. Some plate-like features evident in Figure 1c and Figure S2c are imaging effects showing nanorods formed at the lower cross-section portions of the released polymers.

**Figure 1:**
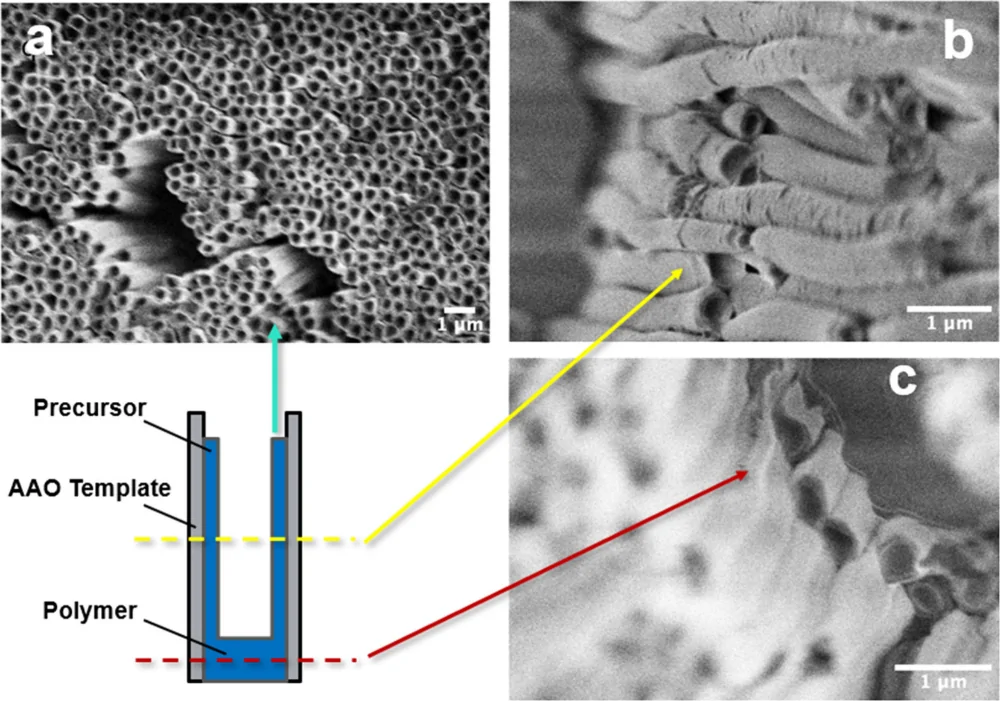
SEM images of the released nanotubes of **PEMA-37k** from AAOC400 templates following imbibition at 493 K for 10 min. Different sections are shown: (a) a top view, (b) a cut about half-height of the templates showing nanotubes, and (c) at the bottom of the templates showing fully infiltrated nanopores.

DSC measurements (Mettler Toledo DSC-822 calorimeter) were performed on both PEMA nanotubes located inside AAOC400 templates and in the bulk samples for comparison. The DSC traces, for **PEMA-7k** and **PEMA-37k**, respectively, are shown in Figure 2a,b and c,d, both at a heating rate of 10 K/min. The results demonstrate a higher *T*_g_ with asymmetric broadening (353 K for **PEMA-7k** and 355 K for **PEMA-37k**) compared to that of the bulk (342 K for **PEMA-7k** and 345 K for **PEMA-37k**). This is attributed to the adsorption effects at the interface that restrict the segmental (and chain) mobility. Consistent with previous molecular dynamics simulations, such adsorption-induced mobility reduction is most pronounced within the first few monomer layers near the wall, effectively raising the local glass temperature relative to that of the bulk.^20,21^

**Figure 2:**
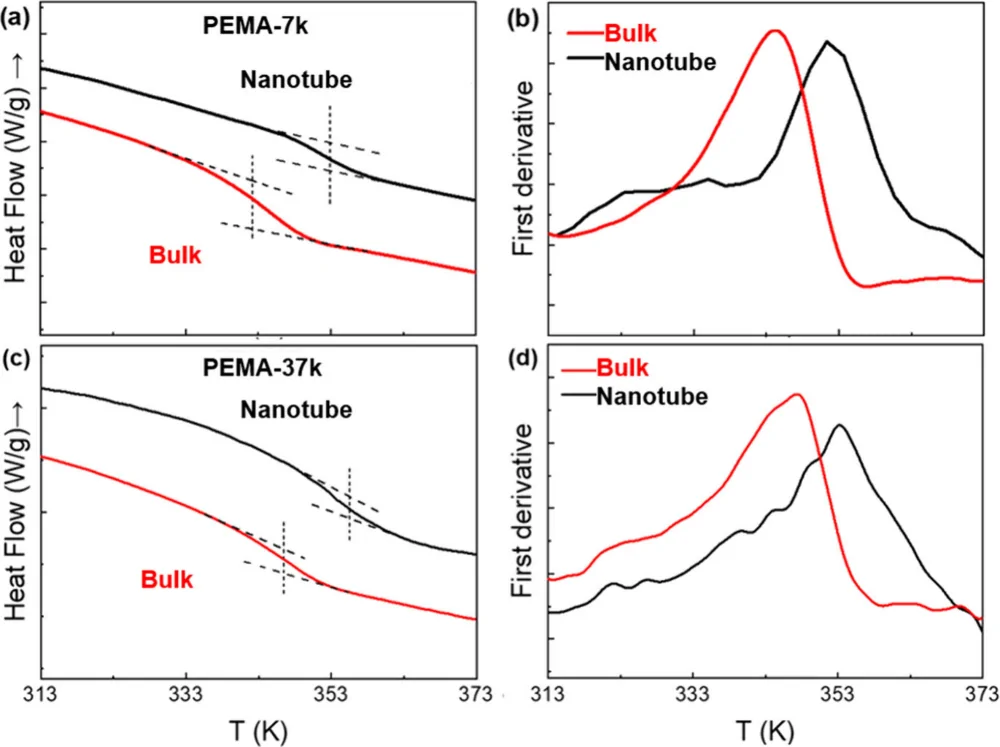
(a, c) DSC curves of nanotubes located inside AAOC400 templates and of the corresponding bulk samples **PEMA-7k** and **PEMA-37k** at heating rates of 10 K/min. (b, d) First derivative of the heat flow with respect to temperature.

The DS study on the dynamics of confined PEMA was made on four different kinds of samples: (i) bulk PEMA, (ii) fully infiltrated PEMA in an AAO template with cylindrical nanopores (AAOC400), (iii) PEMA nanotubes attached to cylindrical AAO nanopores, and (iv) fully infiltrated PEMA in an AAO template with V-shaped nanopores (with 450 nm top diameter, 100 nm bottom diameter, and 2.5 μm depth, AAOV2500) (AAOV2500). Details are provided in the Supporting Information. In all cases, the complex dielectric permittivity ε* = ε′ – *i*ε″, here ε′ is the real and ε″ is the imaginary part, was obtained as a function of frequency ω and temperature *T*. Figure S3 gives representative dielectric loss curves for the bulk- and confined-**PEMA-7k** within cylindrically shaped AAO (AAOC400), in nanotube structures and within conically shaped AAO (AAOV2500). The equivalent capacitor for the system geometry shown in Figure 1 and Figure S2 can be approximated by three capacitors (Figure S4) in parallel as $C_{\mathrm{total}}={\left(\frac{1}{C_{\mathrm{pol}}^{\prime }}+\frac{1}{C_{\mathrm{air}}}\right)}^{-1}+C_{\mathrm{pol}}+C_{\mathrm{AAO}}$ , where *C*_total_ is the total capacitance, *C*_pol_^′^ refers to the polymer capacitance located at the pore bottom, *C*_air_ to the air capacitance located above the polymer, *C*_pol_ refers to the polymer capacitance forming the nanotubes, and *C*_AAO_ to the alumina. The total dielectric function is given by

2 $$\varepsilon_{\mathrm{total}}^{\prime }-i\varepsilon_{\mathrm{total}}^{\prime \prime }=\frac{L\left(\varepsilon_{\mathrm{pol}}^{’}-i\varepsilon_{\mathrm{pol}}^{\prime \prime }\right)}{d_{\mathrm{pol}}+d_{\mathrm{air}}\left(\varepsilon_{\mathrm{pol}}^{\prime }-i\varepsilon_{\mathrm{pol}}^{\prime \prime }\right)}\times \varphi_{\mathrm{air}/\mathrm{pol}}+\left(\varepsilon_{\mathrm{pol}}^{\prime }-i\varepsilon_{\mathrm{pol}}^{\prime \prime }\right)\times \varphi_{\mathrm{pol}}+\left(\varepsilon_{\mathrm{AAO}}^{\prime }-i\varepsilon_{\mathrm{AAO}}^{\prime \prime }\right)\times \varphi_{\mathrm{AAO}}$$

Here, *L* is the template thickness, *d*_pol_ and *d*_air_ are the thickness of the polymer and the air above it, φ_air/pol_, the volume occupied by the air/polymer at the center, φ_pol_ the volume fraction of nanotubes, and φ_AAO_ the volume fraction of AAO (φ_air/pol_ + φ_pol_ is the porosity). After rearrangement, the imaginary part of the dielectric function is given as

3 $$\varepsilon_{\mathrm{total}}^{\prime \prime }=\frac{L\times \varphi_{\mathrm{air}/\mathrm{pol}}}{A^{2}+B^{2}}\times \left(\varepsilon_{\mathrm{pol}}^{\prime }\times B-\varepsilon_{\mathrm{pol}}^{\prime \prime }\times A\right)+\varepsilon_{\mathrm{pol}}^{\prime \prime }\times \varphi_{\mathrm{pol}}+\varepsilon_{\mathrm{AAO}}^{\prime \prime }\times \varphi_{\mathrm{AAO}}$$

where *A* = *d*_pol_ + *d*_air_ × ε_pol_^′^ and *B* = *d*_air_ × ε_pol_^″^. Simulated curves based on eq 3 suggested a minor contribution to the characteristic frequency at maximum loss using the equivalent capacitor model.

DSC measurements in the nanotubes (Figure 2), already revealed an increasing and broadened *T*_g_ relative to bulk. The *T*_g_ increased by 11 K for both **PEMA-7k** and **PEMA-37k** nanotube samples located inside AAOC400 templates. More information on the dynamics can be extracted by DS. PEMA was chosen for this study because of its rich dynamics^36,37^ associated with the liquid-to-glass transformation (α-process with a Vogel–Fulcher–Tammann temperature dependence), the secondary relaxation (β-process with an Arrhenius temperature dependence), and their coalescence to form the αβ-process (also with a VFT temperature dependence). The α-process, the β-process, and the merged αβ processes all occur within a convenient frequency/temperature/pressure window accessible by DS. Earlier studies^36^ as a function of temperature and pressure discussed the origin of the dynamic processes of PEMA through their distinct pressure sensitivities (the apparent activation volumes Δ*V* ^#^) and the ratio *R* = Δ*Ε* ^#^/Δ*Η* ^#^, of activation energies:

4 $$R=\frac{{\left(\partial \ln\tau /\partial \left(\frac{1}{T}\right)\right)}_{V}}{{\left(\partial \ln\tau /\partial \left(\frac{1}{T}\right)\right)}_{P}}$$

It was shown that the α-process bears both intra- and intermolecular contributions. As such, it is controlled both by temperature and volume. However, based on the value of the dynamic ratio Δ*E* ^#^/Δ*H* ^#^ between the two, it is temperature that exerts the stronger influence. The β-process, on the other hand, is mainly of intramolecular origin in agreement with earlier NMR results.^37^ Furthermore, the apparent activation volume, Δ*V* ^#^, revealed that the mixed (αβ)-process (at high temperatures/frequencies) is the structural relaxation, implying that it presents characteristics of a segmental process. The same conclusion was reached^36^ from the values of the ratio of activation energies, Δ*E* ^#^/Δ*H* ^#^, with approximate values of 0.60, 0.90 and 0.68, respectively, for the α-, β- and (αβ)-processes. This value for the (αβ)-process suggests that, while it is controlled both by temperature and volume, the former has the greater influence.

Dielectric loss curves for the different confining geometries are shown in Figures S5 and S6 for the **PEMA-37k** and **PEMA-7k**, respectively. The loss curves depict the three main relaxation processes of PEMA: the β-, α-, and (αβ)-processes. Another process (the “slow” process) exists under the ionic conductivity contribution. The results show that PEMA nanotubes exhibit noticeably broader peaks across all three processes compared to the other types of confinement. To quantify the extent of the broadening, the low- and high-frequency slopes for the α-process were extracted using the HN equation, and plotted as a function of temperature in Figure S7, Supporting Information. For **PEMA-7k**, the low frequency slope (*m*) of the nanotube sample decreased from 0.53 in the bulk to 0.45 in the nanotubes. This effect is more pronounced in **PEMA-37k**, with the *m* value decreasing from 0.5 to 0.35. The broadening of the segmental and local dynamics reflects the wider distribution of relaxation times, resulting from a combination of adsorbed and free chain segments in the vicinity of the pore walls. Simulations of polymer melts under nanoconfinement have demonstrated that such broadening originates from the coexistence of adsorbed chain segments (e.g., loops and trains) with severely slowed dynamics and free segments with higher mobility, leading to a distribution of relaxation times that extends over several decades.^17,30,33–35^

The characteristic relaxation frequencies for all processes are shown in Figure 3 and Figure 4, for **PEMA-7k** and **PEMA-37k**, respectively. The temperature dependence of the characteristic frequencies corresponding to the α- and (αβ)-processes followed the Vogel–Fulcher–Tammann (VFT) equation:

5 $$f_{\max}=f_{0}\exp\left(-\frac{DT_{0}}{T-T_{0}}\right)$$

where *f*_0_ is the characteristic relaxation frequency at maximum dielectric loss corresponding in the limit of very high temperatures, *D* is a dimensionless parameter and *T*_0_ is the “ideal” glass temperature located below *T*_g_. On the other hand, the β-process conforms to an Arrhenius dependence as

6 $$f_{\max}=f_{0}^{\#}\exp\left(-\frac{E}{RT}\right)$$

where *E* is the activation energy. The fitting parameters to the VFT equations are shown in Table 1 and Table 2 for **PEMA-7k** and **PEMA-37k**, respectively. Compared to the bulk, the fully infiltrated PEMA in the cylindrical nanopores exhibits a somewhat higher *T*_g_ and a slower α-process. Similarly, within V-shaped nanopores, which have a slightly larger surface-to-volume ratio than the cylindrical nanopores, there is an increasing *T*_g_. Furthermore, PEMA nanotubes exhibit the highest *T*_g_ among all three cases. This provides additional evidence that the “dead zone” effect is the primary factor slowing both segmental dynamics and the imbibition kinetics during the capillary filling of polymers.

**Figure 3:**
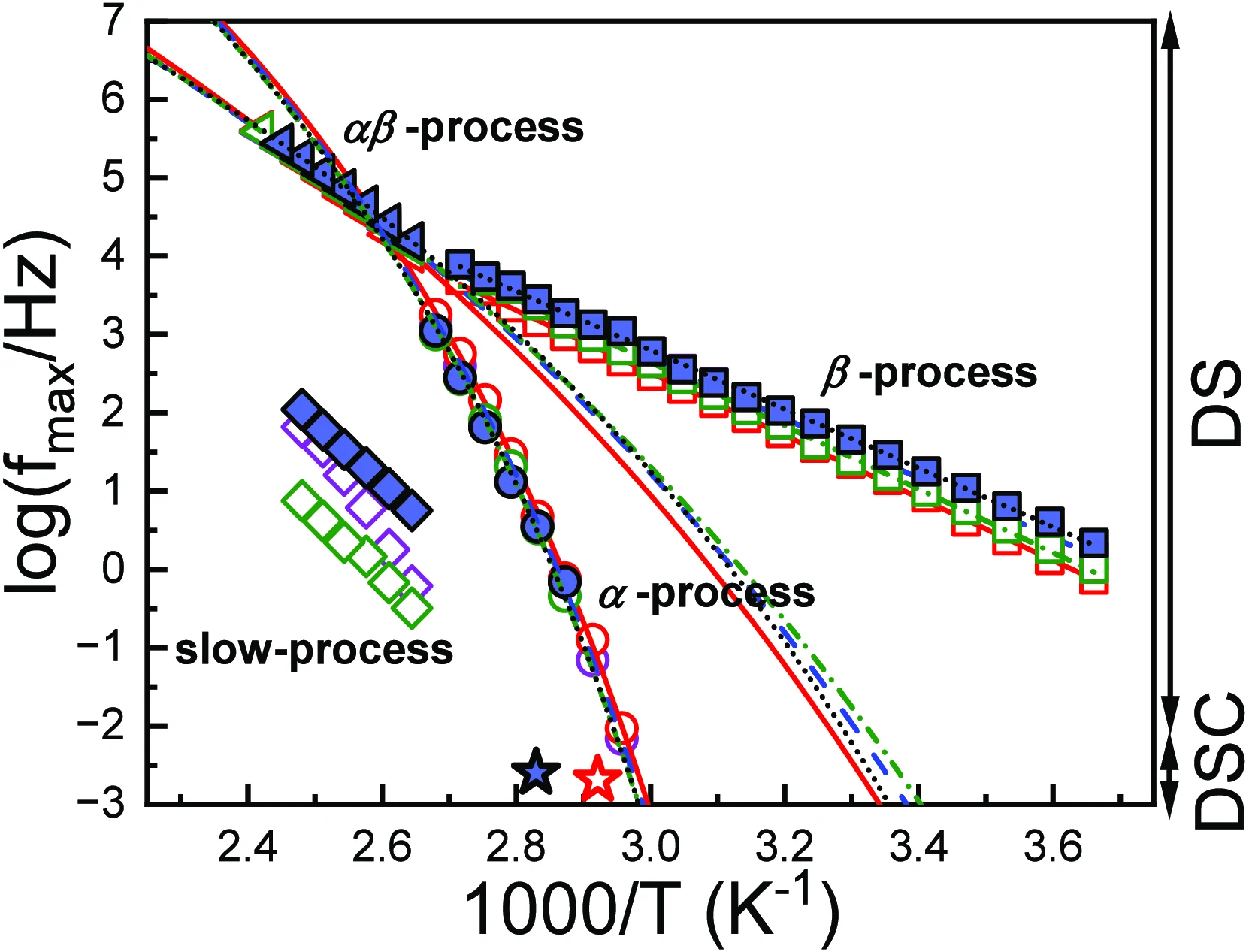
Relaxation frequencies at maximum loss for **PEMA-7k** corresponding to different processes and different confining conditions including nanotubes. From lower temperatures, the β-process (squares), the α- (circles) and the (αβ)- (triangles) processes are shown, together with a slower process (rhombi) under confinement. The data of PEMA-7k nanotubes located inside AAOC400 are shown with blue filled symbols. The bulk data are shown in red, the data of fully infiltrated PEMA in AAOC400 template in magenta and the data of fully infiltrated PEMA in AAOV2500 data in green. The lines are fits to the VFT equation for the slow, (*αβ*)- and α-processes, and to the Arrhenius equation for the β-process. With stars are indicated the bulk and nanotube *T*_g_’s from DSC (at a characteristic time τ ∼ 100 s, *f*_max_ = 1/2πτ_max_).

**Table 1:** Parameters of the VFT Equation for Bulk and Confined PEMA-7k

type of confinement/process	*f*_0_ (s^–1^)	*D*	*T*_0_ (K)	*T*_g_ (K) at τ = 100 s
bulk_α process	3.8 × 10^15^	12.9 ± 3.3	256.5 ± 10.2	334.7
bulk_αβ process	2.2 × 10^14^	25.2 ± 11.3	183.6 ± 29.8	
AAOC400_α process	3.8 × 10^15^ ^a^	12.9^a^	257.2 ± 0.1	335.6
AAOC400_αβ process	4.8 × 10^13^	23.1 ± 3.3	184.8 ± 9.4	
AAOV2500_α process	3.8 × 10^15^ ^a^	12.9^a^	257.7 ± 0.2	336.2
AAOV2500_αβ process	7.3 × 10^13^	25.0 ± 4.7	178.9 ± 12.6	
nanotube_AAOC400_α process	3.8 × 10^15^ ^a^	12.9^a^	257.7 ± 0.3	336.2
nanotube_AAOC400_αβ process	6.3 × 10^12^	17.3 ± 4.6	201.9 ± 16.9	

a Held fixed to the value of bulk.

**Figure 4:**
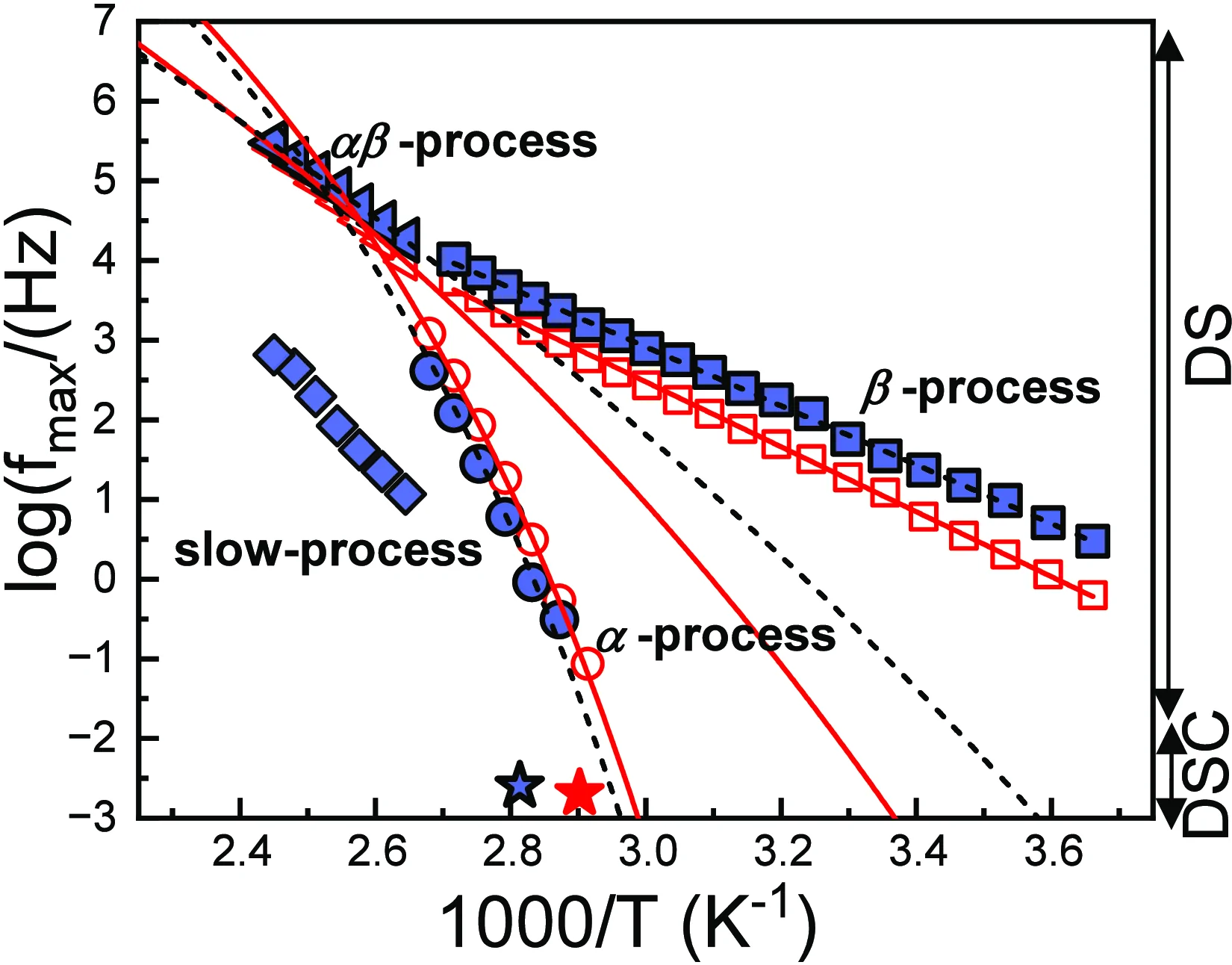
Relaxation frequencies at maximum loss for the bulk and confined **PEMA-37k** corresponding to the different processes in bulk (open symbols) and in nanotubes located inside AAOC400 templates (filled symbols in blue). From lower temperatures, the β-process (squares), the α- (circles) and the (αβ)- (triangles) processes are shown, together with a slower process (rhombi) under confinement. The lines are fits to the VFT equation for the slow, (αβ)- and α-processes, and to the Arrhenius equation for the β-process. With stars the bulk and nanotube *T*_g_’s from DSC.

**Table 2:** Parameters of the VFT Equation for Bulk and Confined PEMA-37k in Nanotubes

type of confinement/process	*f*_0_ (s^–1^)	*D*	*T*_0_ (K)	*T*_g_ (K) at τ = 100 s
bulk_α process	5.8 × 10^15^	13.6 ± 4.2	254.4 ± 13.2	335.3
bulk_αβ process	4.5 × 10^15^	36.2 ± 3.8	161.1 ± 7.0	
nanotube_AAOC400_α process	5.8 × 10^15^ ^a^	13.6^a^	256.7 ± 0.2	338.4
nanotube_AAOC400_αβ process	4.2 × 10^15^	53.5 ± 32.7	124.2 ± 39.0	

a Held fixed to the value of bulk

In addition to the three processes described above in confinement there exists a “slow” process. This process appears under the ionic conductivity and therefore the corresponding frequencies display larger uncertainty. Given the proximity of the process to the measured DSC *T*_g_ we attribute the process to the relaxation of segments in the immediate vicinity of the pore walls (within the adsorbed layer). These results should be compared with recent simulations of polymer (*cis*-1,4-polybutadiene in this case) chains confined between alumina substrates.^24^ There, an effective local glass temperature could be extracted as a function of distance from the substrate. Much higher glass temperatures relative to bulk were found within the first layer (with the length of the repeat unit, *T*_g_ = *T*_g_^bulk^ + 123 K) and within the second layer (a region of about 2 repeat unit lengths exhibited *T*_g_ = *T*_g_^bulk^ + 25 K), while in more distant layers *T*_g_ = *T*_g_^bulk^. In view of the simulations, we attribute the “slow” and the “segmental” process to the relaxation of segments in close proximity to the pore walls and further away, respectively, both located within the adsorbed layer. It is surprising that “dead layer” effects could be detected in the experiments in a nanotube with thickness 15 × *R*_g_. It suggests that the adsorbed layer extends far away from the pore surface. Of key importance here are the configurations of long loops which are formed following polymer imbibition, as revealed from recent studies by the *in situ* nanodielectric spectroscopy of type-A polymers in the same AAO templates.^17,30,33–35^

In summary, the precursor film made during the wetting transition of a polymer in the inner walls of cylindrical alumina nanopores contain adsorbed chains that give rise to a “dead layer”. This is manifested in the bimodal segmental dynamics and the broad distribution of relaxation times. The experimental findings are in excellent agreement with molecular dynamics simulations, which consistently predict that the dead layer exhibits sluggish but finite segmental mobility, a broad relaxation spectrum, and a strong dependence on chain length and confinement, thereby unifying the understanding of polymer dynamics under nanoscale wetting conditions. These features of polymer wetting – especially the effective interfacial thickness, the stability and mechanical integrity of the interface – are of key importance in designing surfaces for lubrication and in coatings.

## Supplementary Material



## Notes

Characteristics of samples and AAO templates; Nanotube preparation; Details on SEM, DSC, and dielectric spectroscopy (PDF)
