# Supplementary material for: “Dead” Layer Dynamics of Poly(ethyl methacrylate) in Nanotubes
Source: ACS Macro Lett. 2026 Jul 22;15(8):1123–8. doi: 10.1021/acsmacrolett.6c00291 (PMC13492377; doi:10.1021/acsmacrolett.6c00291)
Supplement: Supplementary file 1 [file mz6c00291_si_001.pdf]

Supporting Information

"Dead" Layer Dynamics of Poly(ethyl methacrylate) in Nanotubes

Jianwei Zhang,[1,2,5] Jiajia Zhou,[3*] Hans-Jürgen Butt,[1] and George Floudas[4,1*]

[1] Max Planck Institute for Polymer Research, 55128 Mainz, Germany

[2]Faculty of Materials Science and Engineering, South China University of Technology, Guangzhou 510640, China

[3] South China Advanced Institute for Soft Matter Science and Technology, School of Emergent Soft Matter, South China University of Technology, Guangzhou 510640, China

[4] Department of Physics, University of Ioannina, 45110 Ioannina, Greece

[5] National-certified Enterprise Technology Center, Kingfa Science and Technology Co., LTD, Guangzhou 510663, P. R. China

*Corresponding authors: zhouj2@scut.edu.cn, gfloudas@uoi.gr

Outline

1. **Samples and AAO templates**
2. **Contact angle measurements**
3. **Nanotube preparation**
4. **Scanning Electron Microscopy (SEM)**
5. **Differential scanning calorimetry (DSC)**
6. **Dielectric Spectroscopy**

1. Samples and AAO templates. Two poly(ethyl methacrylate) (PEMA) samples were employed to fabricate nanotube structures. Their molecular characteristics are shown in **Table S1**. Self-ordered nanoporous aluminum oxide (AAO) templates with a pore diameter of 400 nm and a uniform pore depth of 100 μm were fabricated following established literature protocols (AAOC400). In addition to nanotubes we investigate the segmental dynamics within different confining geometries. For this purpose, V-shaped AAOs were purchased from TopTemplates Technology Ltd. (Shenzhen, China) with a top pore diameter of 450 nm, a bottom pore diameter of 100 nm, and pore depth of 2.5 μm, denoted as V-450-100-2500. Prior to the infiltration process, all AAO templates were annealed in a vacuum oven at 433 K for 12 hours. This annealing step eliminates a substantial portion of hydroxyl (-OH) groups from the surface of the AAO.

Table S1. Molecular Characteristics of the PEMA homopolymers.

Polymer	M_n (kg·mol^{-1})	*Đ*
**PEMA-7k**	7	1.23
**PEMA-37k**	37	2.00

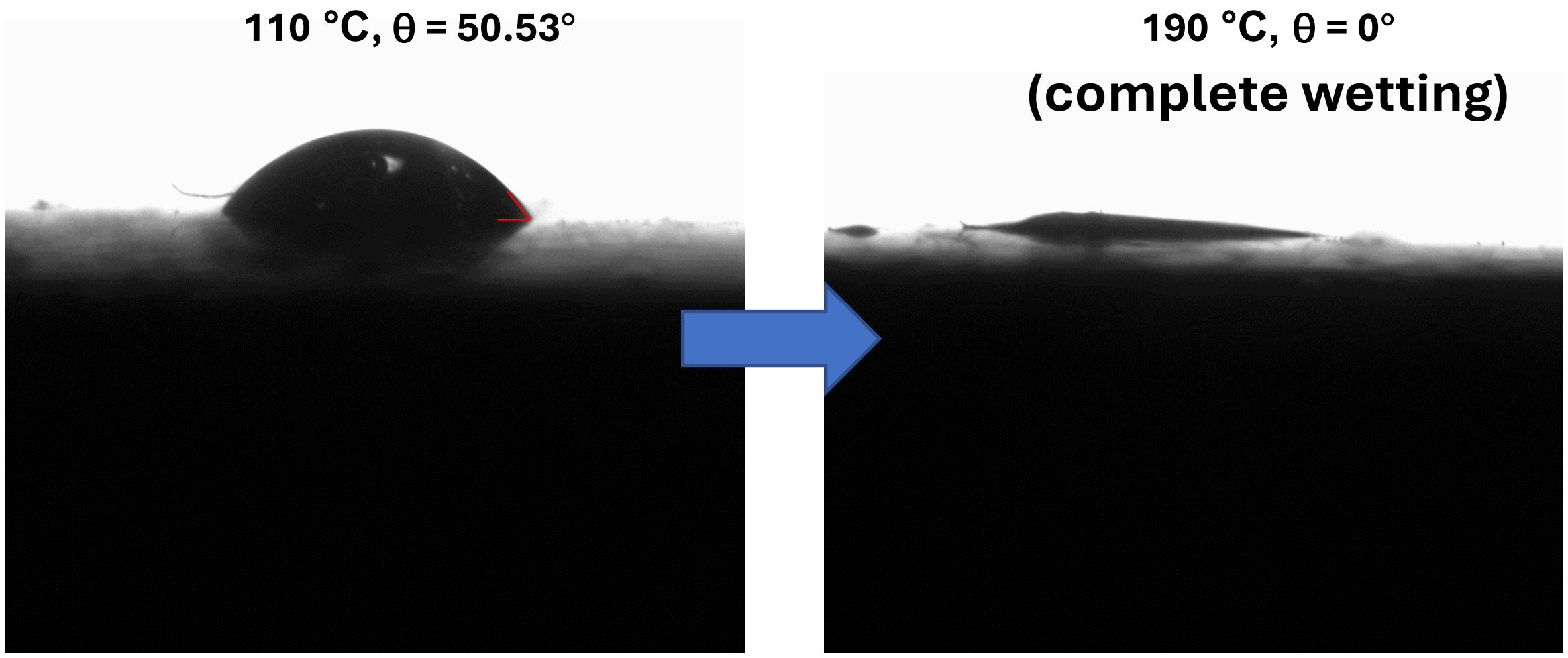

Figure S1. Contact angle measurements of **PEMA-7k** on a flat Al surface at two temperatures. At 463 K there is a complete wetting of the surface.

2. Contact angle measurements. The advancing contact angle of the PEMA was measured by placing nearly spherical polymer particles of ~1 mm diameter onto the substrate. To form spherical particles, around 1 mg of the polymer was placed on a soot-templated superamphiphobic surface at 383 K by following known procedures. Subsequently, the polymer sphere was transferred onto an electropolished Al disk coated by a thin native oxide layer at the same temperature. The latter comprises a flat model surface that mimics the AAO surface. The substrate was heated to 463 K and the advancing contact angle of the polymer melt was measured during spreading with a commercial goniometer (OCA35, DataPhysics). The experiment is shown in **Figure S1**. Complete wetting is obtained with the formation of a macroscopically thick film following heating from 383 K (where the contact angle is at 50°) to 463 K (contact angle of 0°). At this temperature the adhesive forces between the liquid and the substrate are significantly stronger than the cohesive forces within the liquid."

3. Nanotube preparation. We systematically optimized the temperature and time parameters for the two PEMA homopolymer infiltration using the template wetting method. Firstly, a PEMA film was placed on top cylindrically-shaped AAO templates with 400 nm diameter and 100 µm depth nanopores (AAO C400), and the assembly was transferred to a preheated oven for vacuum annealing. The optimal temperature and wetting time for the formation of nanotubes were determined as T=463 K (i.e. T_g+128 K) and 5 minutes for **PEMA-7k**, and T=493 K (i.e. T_g+158 K) and 10 minutes for PEMA-37k, respectively (**Figure S1**).

Subsequently, samples were rapidly cooled to room temperature to preserve the nanostructures. For the DS measurements the nanotubes were not released from alumina. For the SEM characterization, the AAO templates were dissolved using a 5 wt% NaOH aqueous solution, yielding free-standing PEMA nanotubes supported by the residual bulk film.

4. Scanning Electron Microscopy (SEM). The polymer nanostructures were investigated using an SU8000 scanning electron microscope (SEM). The nanotube structures of PEMA were evidenced by analyzing both the top-view and cross-sectional images at different heights. **Figure S2** depict free-standing **PEMA-7k** fibers following AAO removal. In some cases, the nanotubes broke at different height in the process of removing the AAO template. The thickness of the precursor film evidenced by the nanotube wall thickness was around 100 nm. Additional SEM results of PEMA-7k at 400 nm and 65 nm templates are shown in **Figures S3 & S4**.

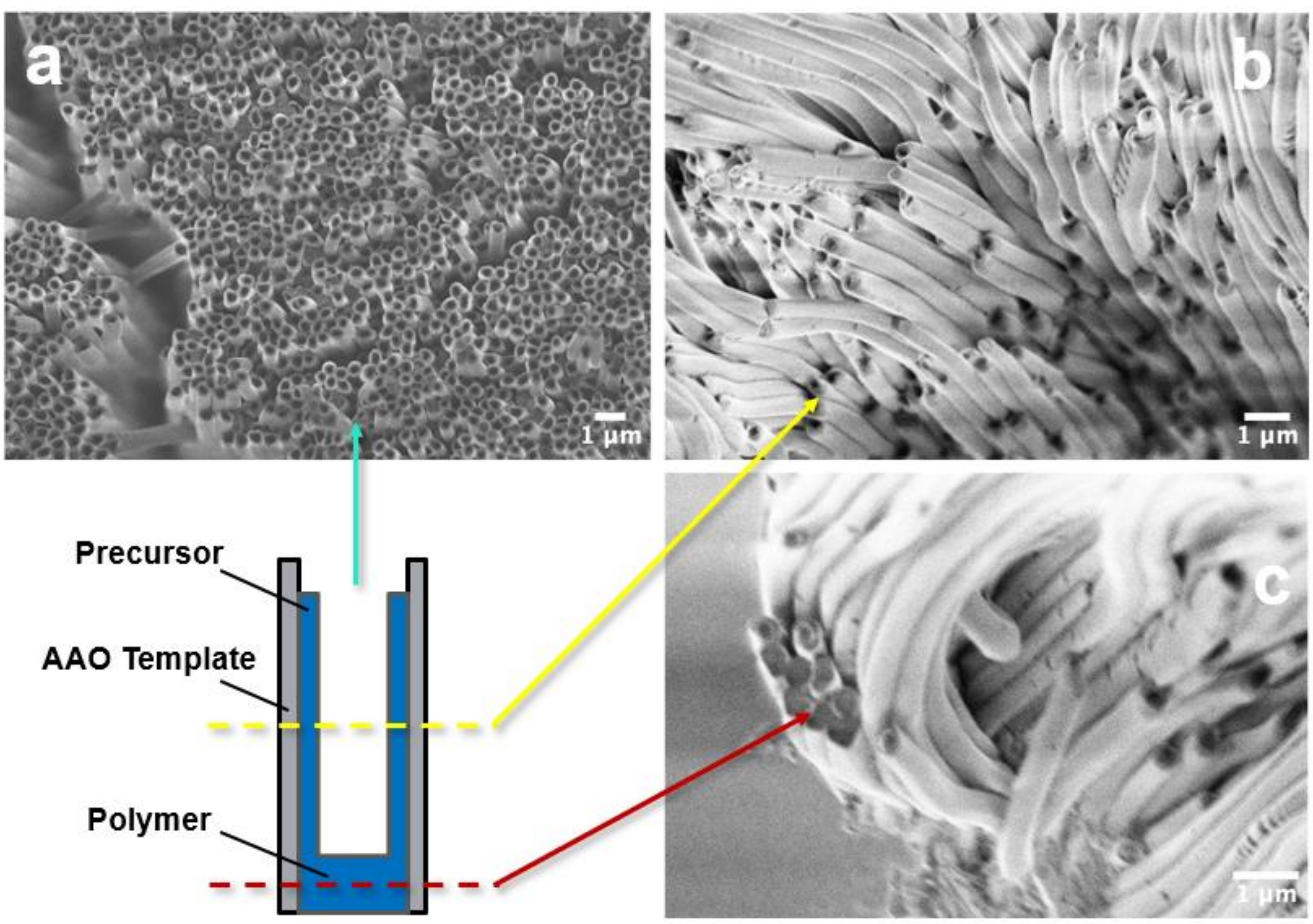

Figure S2. SEM images of the released *nanotubes* of **PEMA-7k** following imbibition at 463 K for 5 minutes in cylindrically-shaped AAO templates with 400 nm in diameter nanopores. Different sections are shown: (a) a top view, (b) a cut about half-height of the templates showing nanotubes, and (c) at the bottom of the templates showing fully infiltrated nanopores.

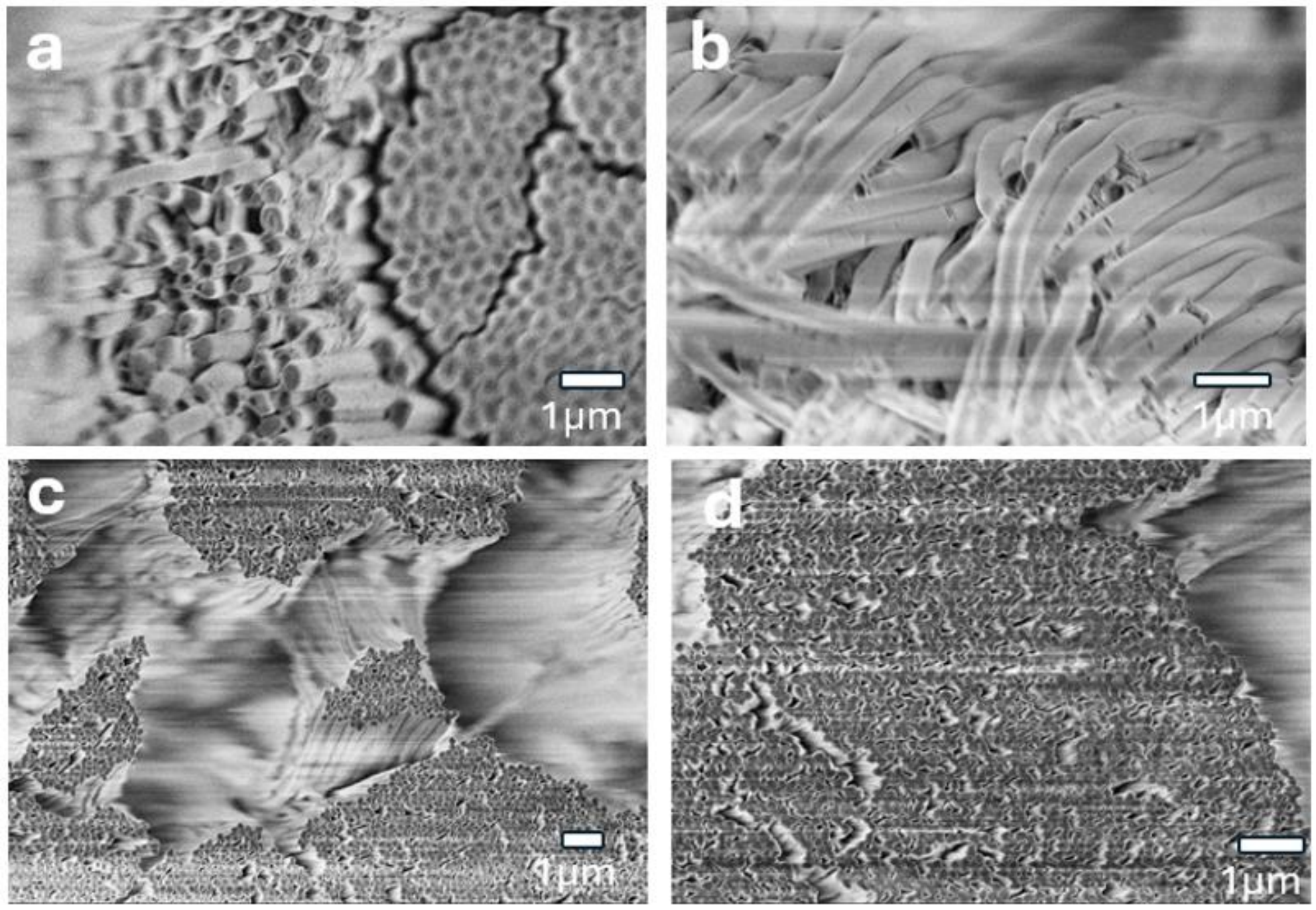

Figure S3. SEM images of the released *nanorods* (top views) of **PEMA-7k** following imbibition at 473 K for 2 minutes (a,b) and 5 minutes (c,d), respectively, in cylindrically-shaped AAO templates with nanopores of 400 nm in diameter.

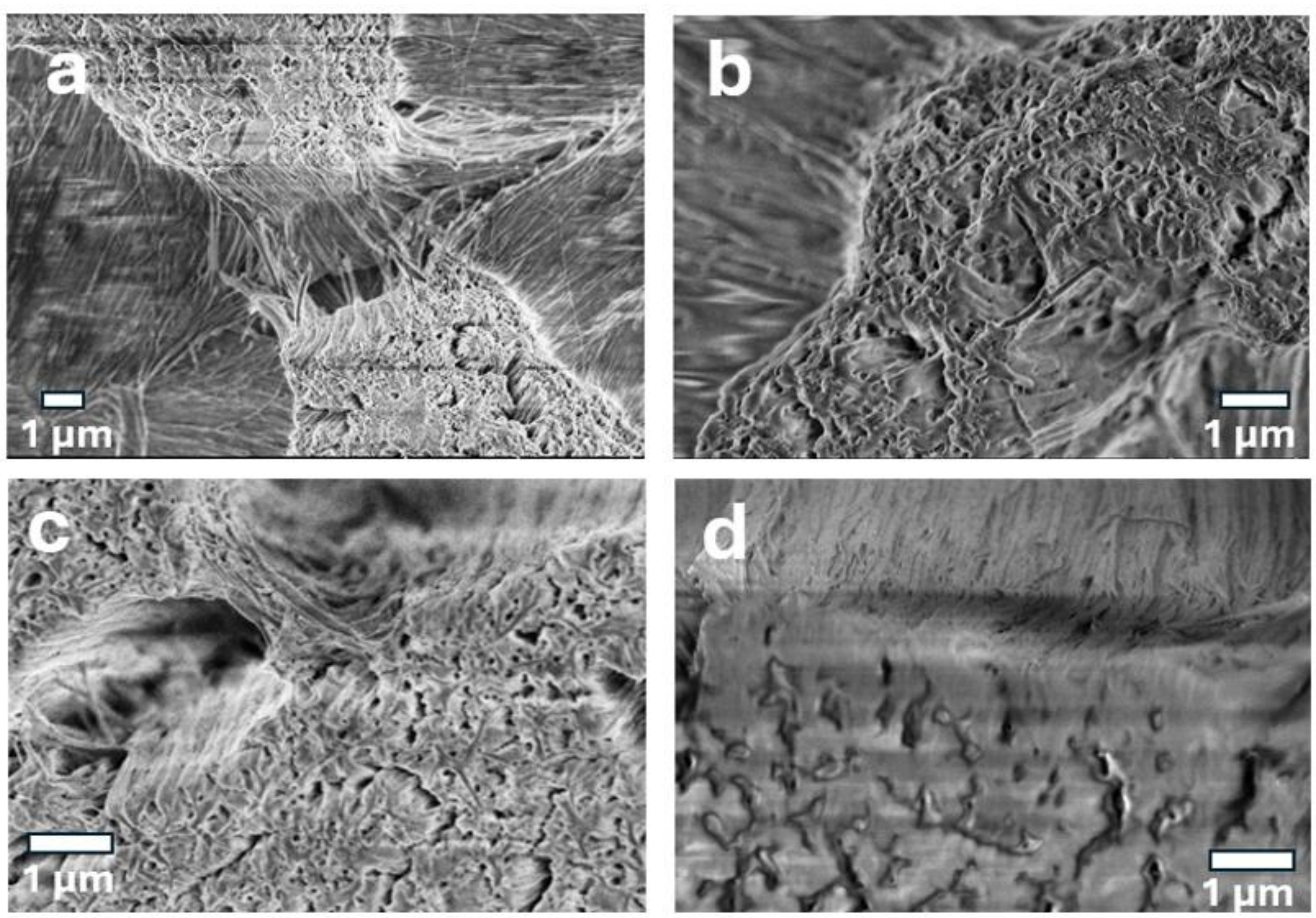

Figure S4. SEM images of the released *nanorods* (top views) of **PEMA-7k** following imbibition at 463

K for 2 minutes (a,b) and 5 minutes (c,d) in cylindrically-shaped AAO templates with nanopores of 65 nm in diameter.

5. FTIR spectroscopy.

Attenuated Total Reflection (ATR) was employed (Bruker Platinum ATR) together with the TENSOR series FT-IR spectrometer. In ATR the intensity the IR light interacts with the first few microns of the sample, allowing for an ATR FT-IR spectrum. The depth of penetration depends on the crystal material (in the range from 0.5 to 4 μm). Three measurements were made at ambient temperature. First, the bulk **PEMA-37K** film, the empty AAO templates and the **PEMA-37k** nanotubes within the AAO templates. The ATR-FTIR spectrum of PEMA (**Figure S5)** show the main characteristic absorption bands [1]: 2990−2950 cm^{-1} (C-H stretching of CH_2 and CH_3 groups), 2940−2850 cm^{-1} (aliphatic C-H stretching), 1720−1730 cm^{-1} (strong ester carbonyl C=O stretching), 1485−1446 cm^{-1} (CH_2 and CH_3 bending/deformation), 1390−1370 cm^{-1} (CH_2 symmetric bending), 1265−1235 cm^{-1} (ester C-O stretching), 1190−1140 cm^{-1} (C-O-C stretching of ester group), and 1140−1020 cm^{-1} (ester related vibrations). The spectrum of AAO shows few weak vibrations as follows [2]: Alumina O-H bands around 3400 cm^{-1} and 1630 cm^{-1} followed by a broad peak at ~ 1100 cm^{-1} from Al-O-H bending vibrations to increased absorption below ~ 800 cm^{-1} due to Al-O vibrations. The spectrum of the **PEMA-37 K** nanotubes located inside AAO templates is a superposition of the characteristic bands of PEMA with the broad bands of AAO [3]. For example, the spectrum exhibits strong stretching bands at 2990−2950 cm^{-1} and 2940−2850 cm^{-1}, the ester stretching at 1720 cm^{-1} followed by CH_2 and CH_3 bending/deformation at 1485−1446 cm^{-1} and by ester stretching at 1265−1235 cm^{-1}, C-O-C ester stretching at 1190−1140 cm^{-1} and ester vibrations at ~1140−1020 cm^{-1} on top of the broad AAO O-H bands at ~3400 cm^{-1} and at ~ 1100 cm^{-1} from the Al-O-H bending vibrations. This is understandable as the penetration depth of the evanescent wave is a few micrometers, i.e. longer than the nanotube wall thickness (~ 100 nm).

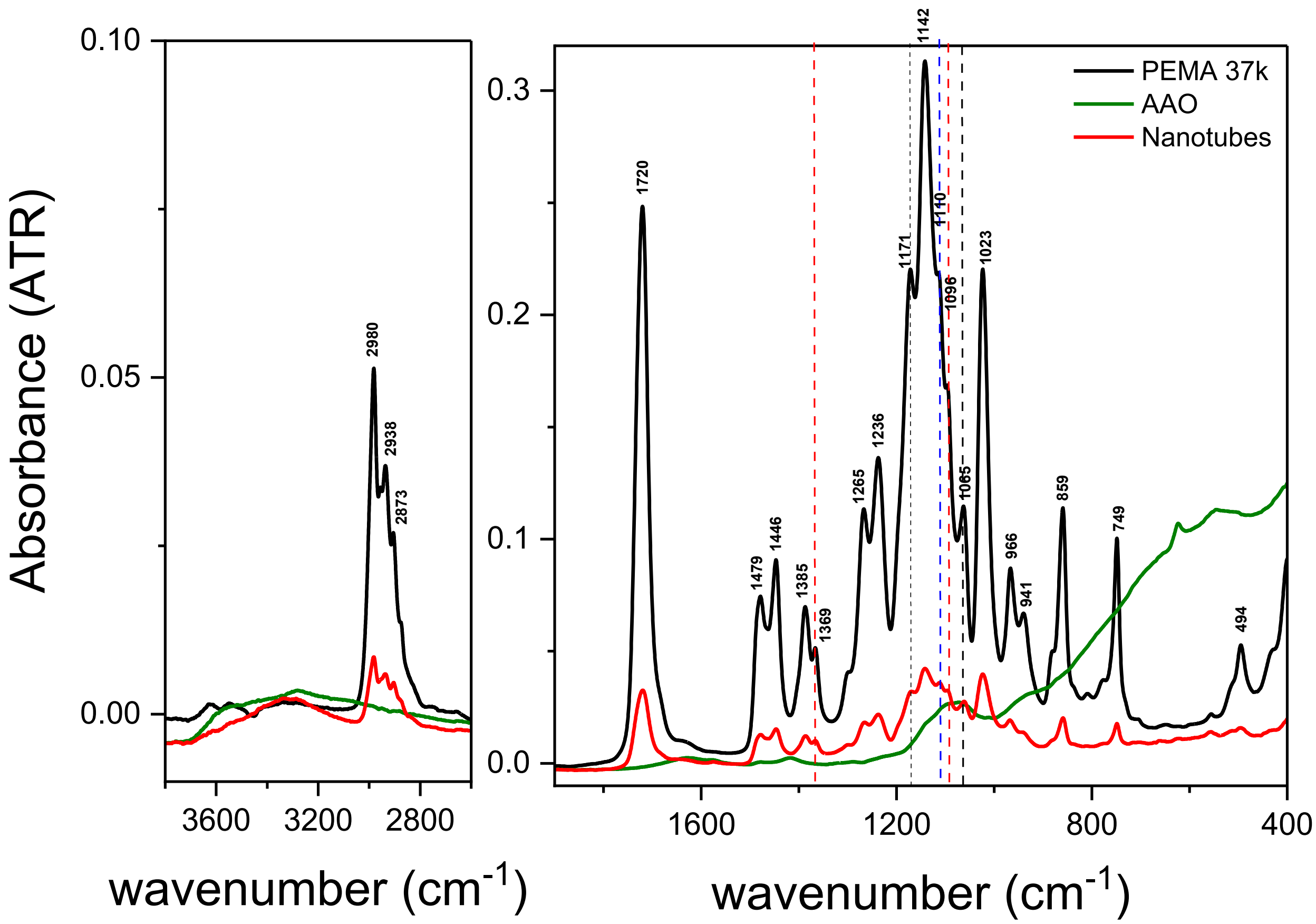

Figure S5. ATR spectra of empty AAO templates (green), **PEMA-37k** bulk film (black line) and **PEMA-37 K** *nanotubes* located inside AAO templates (red line).

6. Differential scanning calorimetry (DSC). DSC measurements were performed on both PEMA nanotubes and in the bulk samples for comparison. Measurements were made with a Mettler Toledo DSC-822 calorimeter. The PEMA nanotube samples were prepared as powders by keeping the AAO template. During the measurement, an empty AAO template with the same mass was used as a reference.

7. Dielectric Spectroscopy. The DS study on the dynamics of confined PEMA was made on four different kinds of samples: (i) bulk PEMA, (ii) fully infiltrated PEMA in an AAO template with cylindrical nanopores (AAOC400), (iii) PEMA nanotubes attached to the cylindrical AAO nanopores, and (iv) fully infiltrated PEMA in an AAO template with V-shaped nanopores (with 450 nm top diameter, 100 nm bottom diameter, and 2.5 µm depth, AAOV2500) (AAOV2500). For each sample, the measurement was

performed at different temperatures in the range of 273-408 K, at atmospheric pressure, and for frequencies in the range from 10^{-2} to 10^{7} Hz using a Novocontrol Alpha frequency analyzer. In all cases, the complex dielectric permittivity $\varepsilon^* = \varepsilon' - i\varepsilon''$, where ε' is the real and ε'' is the imaginary part, was obtained as a function of frequency ω and temperature T. The analysis of the DS curves was based on summation of the empirical Havriliak and Negami (HN) equation:

$$\varepsilon^*(\omega, T, P) = \varepsilon_\infty + \sum_k \frac{\Delta\varepsilon_k(T,P)}{[1+(i\cdot\omega\cdot\tau_{HN,k}(T,P))^{m_k}]^{n_k}} + \frac{\sigma_0(T,P)}{i\varepsilon_0\omega} \qquad (1)$$

where $\Delta\varepsilon_k$ is the dielectric strength, $\tau_{HN,k}$ is the H-N characteristic relaxation time, m_k, n_k (with limits $0.2 < m_k$, $mn_k \leq 1$) describe, respectively, the symmetrical and asymmetrical broadening of the distribution of relaxation times and the index k indicates the process under investigation. At lower frequencies, the dielectric loss sharply rises due to conductivity contribution as $\sigma_0/\varepsilon_0\omega$, where σ_0 is the dc-conductivity and ε_0 (= 8.854×10^{-12} F·m^{-1}) is the permittivity of free space. From $\tau_{\mathrm{HN},k}$, the relaxation times at maximum loss, τ_{max}, were obtained analytically from the HN equation as follows

$$\tau_{max,k} = 1/2\pi\, \mathrm{f}_{\mathrm{max},k} = \tau_{HN,k}\left[sin\left(\frac{\pi m_k n_k}{2(1+n_k)}\right) / \, sin\left(\frac{\pi m_k}{2(1+n_k)}\right)\right]^{1/m_k} \qquad (2)$$

These relaxation times correspond to the relaxation times of the segmental processes. Except for the measured ε'', the derivative of the real part of the dielectric permittivity, ε', ($\frac{\mathrm{d}\varepsilon'}{\mathrm{dln}\omega} \approx -(2/\pi)\varepsilon''$) applicable for broad peaks was used in the analysis of the dynamic behavior. **Figure S6** gives representative dielectric loss curves for the bulk PEMA and confined PEMA within cylindrically shaped AAO (AAOC400), in nanotube structures and within conically-shaped AAO (AAOV2500).

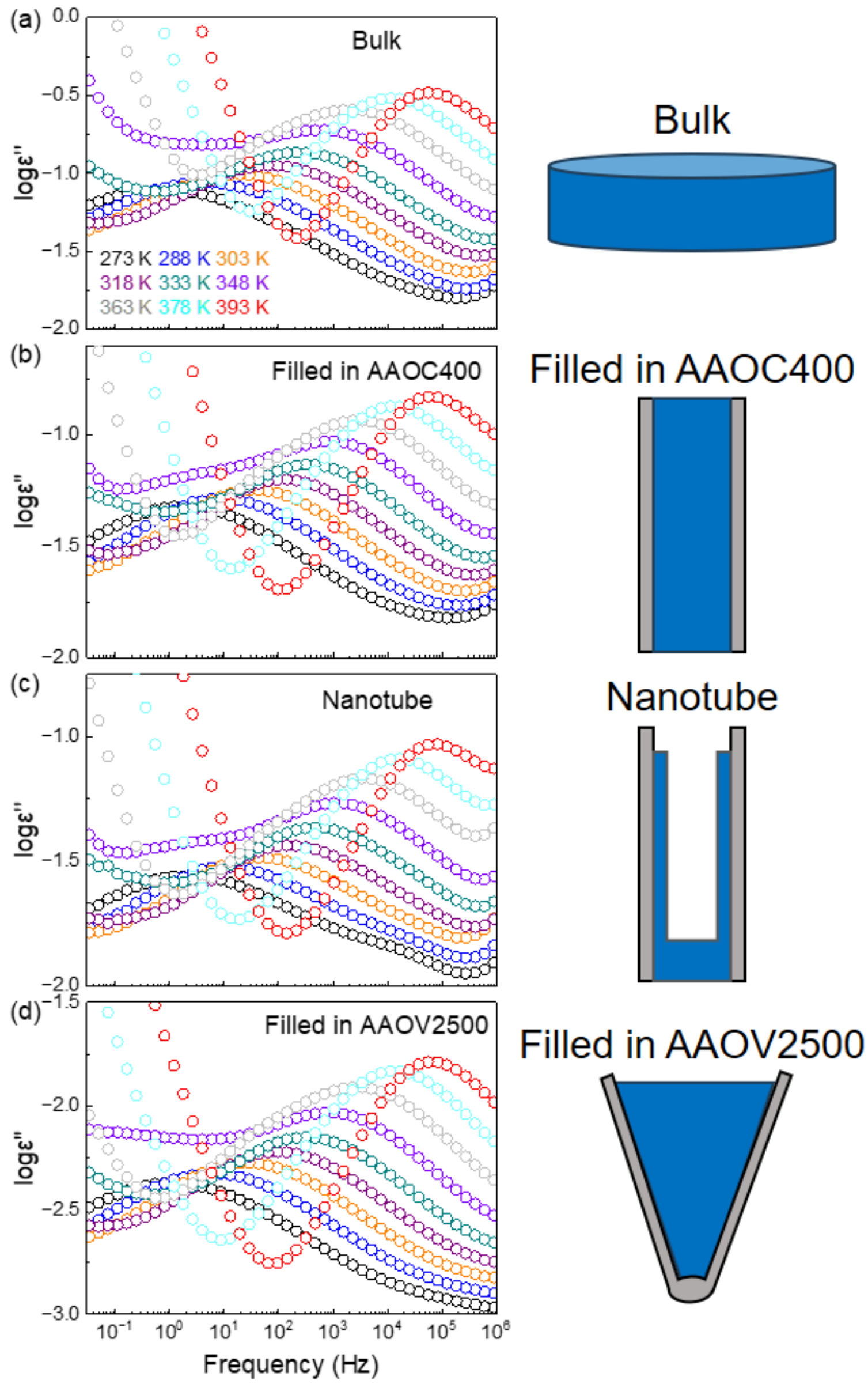

Figure S6. Dielectric loss curves of (a) bulk **PEMA-7k**, (b) fully infiltrated **PEMA-7k** in AAOC400, (c) **PEMA-7k** nanotubes located inside AAOC400, and (d) fully infiltrated **PEMA-7k** in AAOV2500.

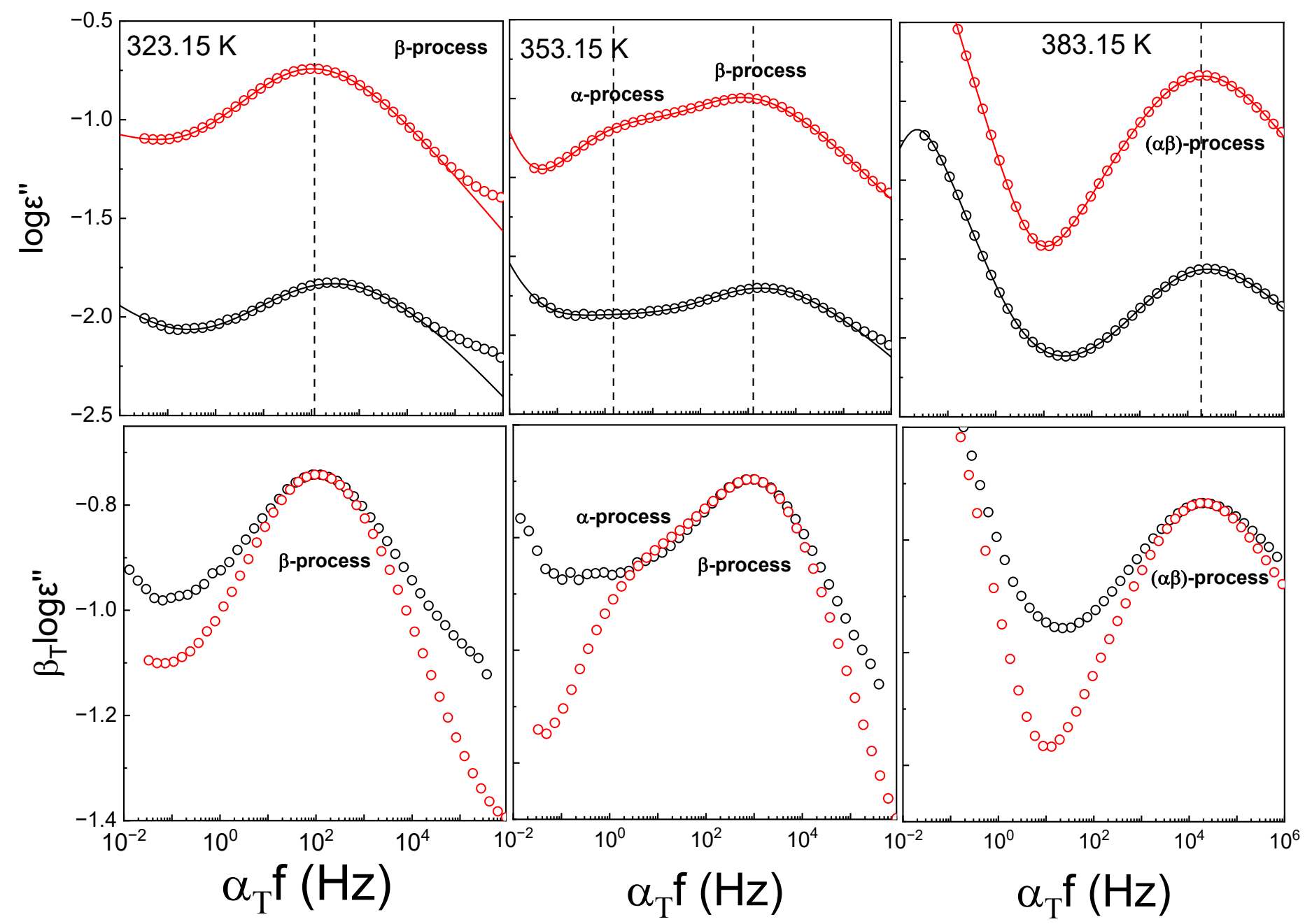

Figure S7. Dielectric loss curves of **PEMA-37k** samples in the bulk (in red) and in nanotubes located inside AAOC400 templates with (a) *β*-, (b) *α*- and *β*-, (c) (*αβ*)- processes, and normalized curves with (d) *β*-, (e) *α*- and *β*-, (f) (*αβ*)- processes.

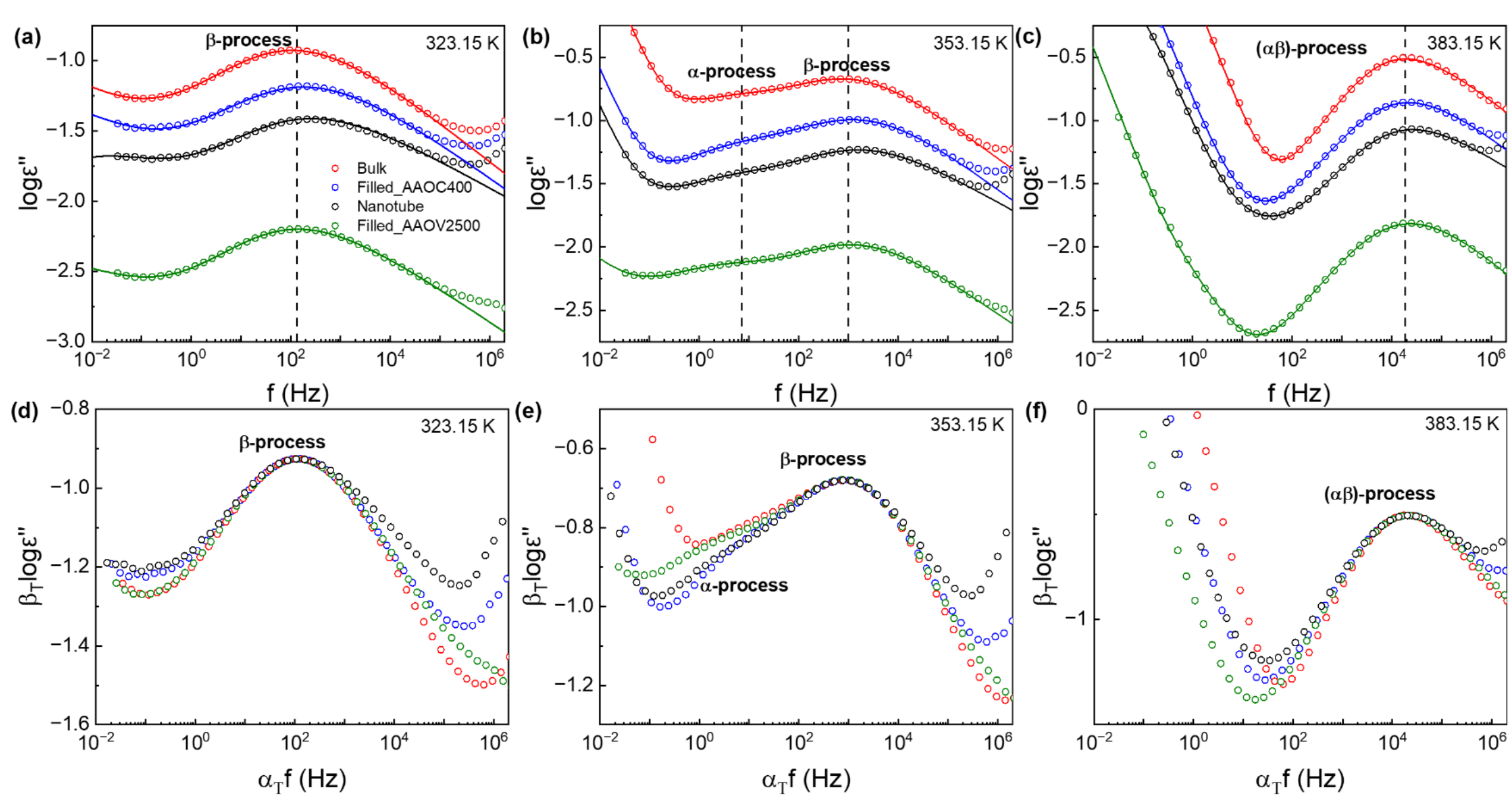

Figure S8. Dielectric loss curves of **PEMA-7k** samples in the bulk (red), in fully infiltrated AAOC400

(blue), in fully infiltrated AAOV2500 templates (green), as well as nanotubes inside AAOC400 templates with (a) β-, (b) α- and β-, (c) ($\alpha\beta$)- processes, and normalized curves with (d) β-, (e) α- and β-, (f) ($\alpha\beta$)-processes.

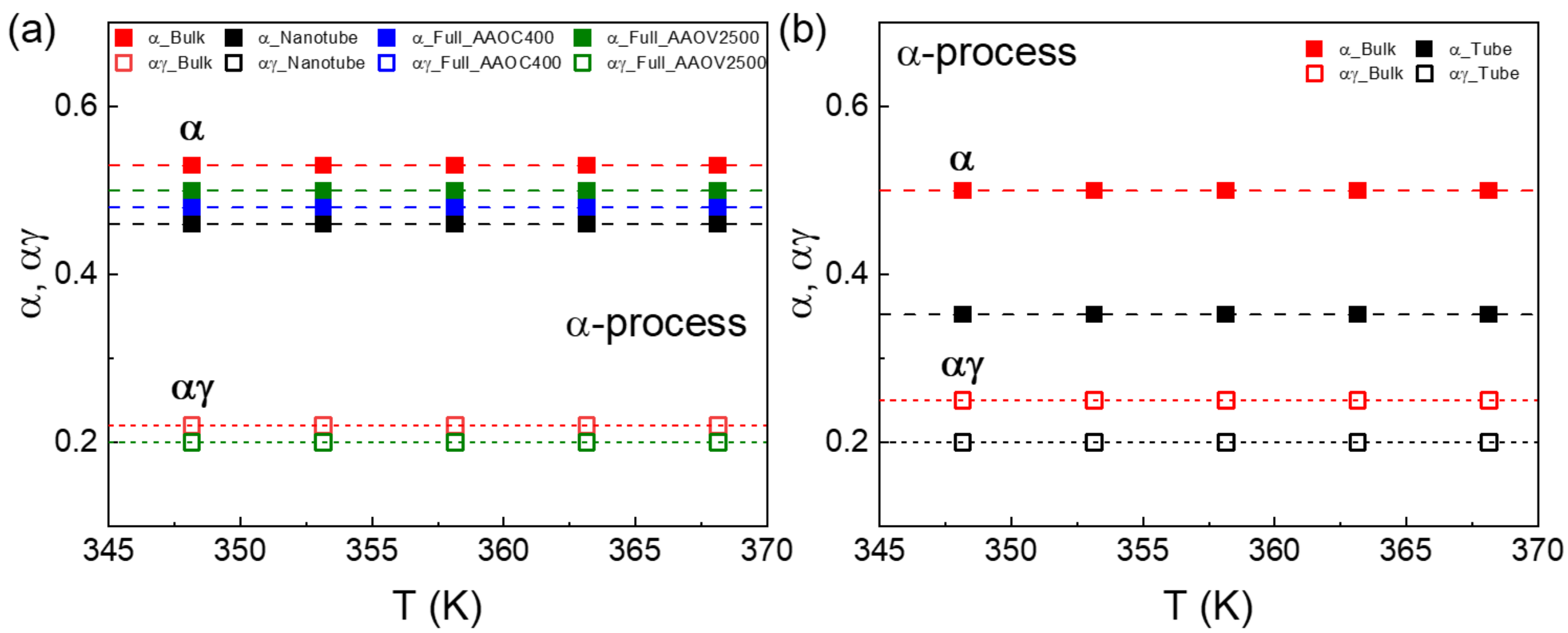

Figure S9. HN shape parameters of the α- process for (a) **PEMA-7k** and (b) **PEMA-37k**. The symbols indicate the polymer in the bulk (in red), polymer in fully infiltrated AAOC400 (in blue), polymer in fully infiltrated AAOV2500 templates (in green), as well as nanotubes inside AAOC400 templates (in black).

References

[1] Abdelrazek, E.M.; Abdelghany, A.M.; Oraby, A.H.; Asnag, G.M. Investigation of Mixed Filler Effect on Optical and Structural Properties of PEMA Films. *International Journal of Engineering & Technology* IJET-IJENS **2012**, Vol:12 No:04.

[2] Balan, E.; Theoretical Infrared Spectra of OH Defects in Corundum (α-Al2O3) *Eur. J. Mineral.,* **2020**, *32*, 457–467.

[3] Sai Prasanna, C.M.; Suthanthiraraj, S.A. Investigations of Zinc Ion Dissociation in Gel Polymer Electrolytes Based on Poly(vinyl chloride) and Poly(ethyl methacrylate) Blend on the Addition of Two Different Ceramic Nanofillers. *Journal of Inorganic and Organometallic Polymers and Materials*, **2019**, *29*,483–501.
